# Using online spaces to recruit Kenyan queer womxn and trans men in restrictive offline settings

**DOI:** 10.1186/s13690-022-00824-3

**Published:** 2022-03-14

**Authors:** Stephanie Haase, Virginia Zweigenthal, Alex Müller

**Affiliations:** 1 School of Public Health and Family Medicine, University of Cape Town, Cape Town, South Africa; 2 Department of Medical Ethics and History of Medicine, Universitätsmedizin Göttingen, Göttingen, Germany; 3 Gender Health and Justice Research Unit, University of Cape Town, Cape Town, South Africa

**Keywords:** Sexual and gender minorities, Kenya, Methodology, Online, Restrictive context

## Abstract

**Background:**

Understanding and addressing healthcare and service delivery inequalities is essential to increase equity and overcome health disparities and service access discrimination. While tremendous progress has been made towards the inclusion of sexual and gender minorities in health and other research, gaps still exist. Innovative methods are needed to close these. This case study describes and reflects on using online-based data collection to ascertain sexual health decision-making and health service utilisation among Kenyan queer womxn and trans men.

**Methods:**

**Case study**

The study used a mixed-methods approach in two phases with triangulated quantitative and qualitative elements. Both elements used web-based technology to gather data.

**Results:**

Using online spaces to recruit and collect data from queer womxn and trans men exceeded expectations. A total of 360 queer womxn and trans men responded to the digitally distributed survey, and 33 people, queer womxn and trans men, as well as key informants, participated in the interviews, which were primarily conducted on Zoom and Skype. The case study analyses the risks and benefits of this approach and concludes that online sampling approaches can mitigate risks and enable effective and safe sampling of a marginalised group in a restrictive legal setting: Kenyan queer womxn and trans men.

**Conclusion:**

Using online spaces when researching marginalised populations could effectively overcome risks around stigma, discrimination and violence. It could be an effective way to understand these populations’ healthcare needs better. Factors contributing to success included building trusting relationships with key members of the community, strategic and opportune timing, a nuanced understanding of the mobile landscape, and carefully chosen safety and security measures. However, it should be noted that conducting research online could increase the risk of further marginalising and excluding those without access to web-based technology.

## Background

The inclusion and recruitment of hard-to-reach populations in research can seem difficult for various reasons. Access to marginalised populations might be especially challenging during the ongoing COVID-19 pandemic. This case study describes the effective and safe recruitment of Kenyan queer womxn^1^ and trans men reached in a restrictive, hostile context using online methods. With access to the internet continuously expanding worldwide, such approaches could be applied to other marginalised populations in various contexts.

### Research on the healthcare needs of sexual and gender minority people

Understanding and addressing healthcare and service delivery inequalities is important to overcome discrimination and increase equity [4]. While progress has been made towards the inclusion of sexual and gender minorities in health and other research, gaps still exist [14, 28]. Less than 3% of all published research from 2000 to 2015 dealt with the health concerns of people who identify as sexual and gender minorities, and those published articles that did, often did not report on population-specific factors such as homophobia or outness – disclosing one’s sexual orientation or gender identity (van Eeden-Moorefield et al. 2018). Even a general understanding of the demographics and information on health, economic and social factors that influence the health of sexual and gender minority people are frequently lacking [47]. As most current research on sexual and gender minority health issues focuses on men having sex with men (MSM) and HIV/AIDS, there is consequently little knowledge about ageing sexual and gender minority populations or on health risks and effective prevention of ill-health among sexual minority women [9].

Further, most research on the health of sexual and gender minority populations is conducted in Europe and North America, considerably different contexts compared to the global south. For example, only eight of 76 articles reviewed in a recent systematic review of sexual orientation- and gender identity-motivated violence were from countries outside the E.U., the U.S. or Canada. Of those eight, two were from African countries (Rwanda and Cote d’Ivoire) (Blondeel et al. 2018).

### Sampling sexual and gender minority populations in restrictive settings

Sexual and gender minorities can be a hard-to-reach and vulnerable population, as they tend to be hidden,^2^ due to fear from being exposed – outed, discriminated against, or otherwise violated. Disclosing one's sexual orientation and gender identity can increase one's risk of experiencing stigma, discrimination, or violence, especially in contexts where sexual and gender diversity is considered taboo or where same-sex sexuality is criminalised by law.

This can result in sexual and gender minority people being difficult to identify and include in research purposefully. Access, however, is not the only barrier. Gaining the participants’ confidence and interest is important for participation. Given the history of discrimination against sexual and gender minorities, including in healthcare [48], sexual and gender minority people might be reluctant to engage with the healthcare system and health researchers [3]. Thus, identifying and recruiting participants for research regarding their needs can be difficult,special attention should be given to ensure that there is minimal risk for all participants, and that representatives from the communities should be engaged from the beginning of the research process [37]. Additionally, building a trusting relationship and dealing with the participants’ concerns of being exposed are considered a high priority, especially when the research area dealt with sensitive topics [6, 18].

Other difficulties in sampling include participants' lack of time to participate or transportation to the data collection site [18]. Addressing these barriers can have significant cost and time implications. Building relationships with people from the communities to be included and involving them in the research development takes time but could have advantageous effects [6].

Understanding the needs of hard-to-reach populations is not just important for the populations themselves; including them in research will facilitate increased knowledge that could potentially affect the burden of disease and gaps in current healthcare provision [18]. Additionally, excluding populations threatens external validity and the ability to generalise research findings [6].

The sampling of sexual and gender minority populations can thus be challenging due to factors such as defining criteria for participation, navigating contexts of stigma and discrimination, and ethical concerns within restricted contexts.

### Definitions

In order to be able to recruit a representative sample, a clear definition of the population to be researched is needed. However, defining sexual orientations and gender identities can be difficult, which could result in an ill-defined research population. It is argued that not defining alternatives to heteronormativity – queerness – dilutes the essence of queerness, as being ill-defined is inherent to its nature [1]. Sexuality and gender and their definitions can be fluid and may change over time ([35, 43]), or individuals may not identify themselves within the categories used [33], regardless of their behaviour. For example, someone exclusively having sex with other womxn may not define themselves as a lesbian or queer person if they have not officially ‘come out’, are hiding their identity or are not familiar with the terminology. This can make sampling difficult, especially if the inclusion criteria may match a participant’s behaviour but not their definition of their sexual orientation or gender identity. Terminology about sexual and gender identity, and thus self-identification is place-, context-, time- and language-specific. For example, whilst the terms ‘lesbian’ and ‘gay’, for example, are widely used in Europe and North America, they do not hold the same identification value in other parts of the world.

### Stigma, fear, discrimination: risks

In evaluating research risks and benefits for participants, research with sexual and gender minority participants is often considered high-risk, as it frequently involves topics on mental health, substance abuse, and identity concerns [31]. These issues are considered by Ethics boards reviewing research proposals. Topics are seen to potentially cause harm when study participants answer questions that might trigger re-traumatisation or other harmful emotions. However, empirical research suggests that this perceived harm is based on unconfirmed assumptions and that there is no evidence that discussing these subjects causes psychosocial or other harm to sexual and gender minority participants [15]. Other studies have shown that answering questions on topics that have been considered high-risk for youth during research processes does not cause more stress than if those topics would be addressed during a medical check-up [16]. Also, as there is little empirical data on what ‘minimal risk’ is – a notion considered in ethics reviews, studies have suggested that ethics bodies frequently have to rely on their subjective judgements and could hence overestimate the likelihood and degree of potential harm [15].

For many sexual and gender minority people, a risk of participating in research is that their sexual orientation and or gender identity could unintentionally be disclosed in or through the research process. This could increase their risk of experiencing violence, stigmatisation and discrimination, especially in settings where consensual same-sex activities are criminalised or where sexual and gender diversity are heavily stigmatised.

Stigmatisation and fear of disclosing one's sexual orientation to a researcher are factors that could complicate sampling [35]. Sexual and gender minority persons who have not shared their identity or orientation with others may not want to disclose their sexual orientation to a researcher [35] or show low interest and distrust [18].

For sexual and gender minority youth, who might need parental permission to participate in research, the risk of parental rejection or neglect is an additional potential risk. Researchers may be required by law or regulations to get consent to participate in sexual orientation and gender identity research ([13, 12, 43]).

### Risk versus agency

While sexual and gender minorities are considered vulnerable populations, sexual orientation or gender identity does not influence understanding and giving informed consent. Participants are vulnerable due to the context they live in [31]. At the same time, participants have agency and know best what risks they could face by participating. Consequently, they are not a vulnerable group as defined in ethics regulations [39]. This was found in a study among sexual and gender minority youth giving self-consent in research on HIV prevention. The participants could accurately reflect on the critical information on risks and informed consent they were provided with and understood that they could refuse participation. Most participants also indicated feeling comfortable participating, claiming ‘*it was their responsibility to make decisions that would affect their health*’ ([3], p. 6).

### Using online approaches to recruit and sample sexual and gender minorities

Innovative processes are needed in recruitment and sampling processes to account for the consequences of the context and its sensitivities in which research is being conducted [33]. One innovative approach, which has become frequently used over the last decade, utilises the internet to recruit sexual and gender minority people for research [18, 19]. By working with populations in online spaces that provide more opportunities to ensure anonymity, some of the challenges of sampling and recruiting sexual and gender minorities could be mitigated. Online approaches have successfully reached marginalised, less visible populations, such as young LGBTQ people [34]. Other advantages include easier access to target groups in general, ease of use in sensitive matters, easier data processing and reduced risk of data loss [27]. The approaches, however, have not always been effective for qualitative research, as McCormack [33] reports, due to participants not attending scheduled interviews and, in some cases, misunderstanding the aims and purposes of the online interaction. Other disadvantages of collecting data online include low response rates, the inability to explain and walk participants through quantitative data collection tools, being an annoyance (frequently disturbing the participants with invitations to participate) and low external validity [27].

Research suggests that, for quantitative data collection methods, findings of self-administered surveys are comparable for paper and web-administered surveys [21]. The internet has also been shown to be an important medium to seek sexual health-related information for MSM [39], especially in the United States. As internet coverage is increasing worldwide, and United Nations bodies suggest that access to the internet should be considered an auxiliary human right, it was postulated that the web could be a valuable medium to be used in different contexts [32].

However, it should be noted that using online approaches and social media to recruit hard-to-reach populations is not without risk: cyberbullying and discrimination can be a concern when using social media as a recruitment tool [42]. Another concern around using online spaces for research, unrelated to the risks for participants, is people submitting the survey multiple times, resulting in skewed results.

The challenges of conducting research online are exacerbated in contexts where same-sex activity is criminalised, and stigma and discrimination against sexual and gender minorities exist. The following case study examines how online sampling approaches can mitigate some of the risks and enable effective and safe sampling of a marginalised group in a restrictive legal setting: Kenyan queer womxn and trans men. A similar approach might also be effective in different contexts with marginalised or at-risk populations.

### Context

In Kenya, the Penal Code criminalises consensual same-sex activity [29] as a felony, with maximum jail sentences of seven years. This negatively affects the lived experiences of sexual and gender minority people, including their access to sexual health information and health services. The criminalisation and the lack of protection against violence, torture, and public humiliation, can negatively impact the well-being of sexual and gender minorities (Human Rights Watch, 2008, [49]. Restrictive laws and policies can be used to justify torture and ill-treatment of sexual and gender minorities, especially by law enforcement agents. This institutionalised form of discrimination exacerbates stigma and prejudice in settings beyond law enforcement [2, 36, 44]. Restrictive laws further degrade dignity, invade people's privacy, create fear and invisibility and '*relegate people to inferior status because of … who they love*’ [22p. 86]. In short, they cause state-sponsored homophobia [44]. Excluding sexual and gender minority’s issues from policymaking further adds to the neglect of their health needs and increases their vulnerability [24, 26].

An example is the HIV/AIDS response in Kenya, where only men who have sex with men (MSM) – a key population – are included in HIV prevention and treatment programmes by both the National AIDS Control Council and the National AIDS & STI Control Programme [23]. Other public sector health efforts (prevention, service provision and research) are heteronormative. Consequently, other sexual and gender minorities remain marginalised in research and care. Little is known about their lived experiences regarding discrimination, stigmatisation, specific healthcare needs, and access to services [17]. In particular, little research exists on queer womxn and trans men – their health status and service needs, and more research is needed to understand the health needs of all sexual and gender minorities in Kenya.

This case study is based on a larger research project on the sexual health and sexual decision-making of queer womxn and trans men in Kenya. Due to the restrictive context, sampling and recruitment were assumed to be difficult. The data were sensitive, as the participants shared personal information about their sexual orientation and gender identity and expression (SOGIE) and their sexual practices. Whilst the former (SOGIE) is not illegal in Kenya, engaging in same-sex activity is. Further, knowledge of one's SOGIE can be used to threaten people with the disclosure of their SOGIE against their will and result in blackmail and extortion or lead to violence. More traditional sampling methods, such as probability sampling of existing databases to reach potential participants, may not have yielded adequate results for this research, due to fear of discrimination and violence among the study participants, or might not have been possible at all, as there are no existing databases or data on queer womxn and trans men. A safer approach needed to be chosen.

Kenya has high mobile and internet penetration: Mobile penetration in Kenya was 94% in December 2017. There were over 33 million mobile data subscribers in the country (Communications Commission of Kenya, 2018). Researchers have suggested that conducting data collection online can ensure higher levels of anonymity and privacy for the participants, hence lowering the risks of participating; while at the same time reaching a potentially higher number of participants [41].

### Aim

This case study explored how online approaches can effectively and safely recruit and collect data from queer womxn and trans men in a restrictive setting that makes using offline approaches more difficult.

### Approach

A mixed-methods approach was used in two phases with triangulated quantitative and qualitative elements to allow for deep insight into the feelings, emotions and experiences that shape sexual health decision making and service utilisation among queer womxn and trans men. It should be empathised that the purpose of this case study was to elaborate on the recruitment of queer womxn and trans men. Recruiting a broad sample was central to the study design. However, the intention was not to be representative but to surface hard-to-reach, under-researched population. As very little is known about the target population’s demographic profile in Kenya, it was not possible to determine whether or not the participants were a true representation of the population. It was assumed that recruiting sufficient numbers of participants would be difficult due to fear of stigma and discrimination, being outed, or distrust of the researcher, or an inability to reach sufficient participants. Alongside the survey and interviews with queer womxn and trans men, interviews were also conducted with other key informants with experience in policymaking or delivering services to this population. However, reaching them was not assumed to be a concern and their recruitment was not explicitly mentioned in this case study.

### Eligibility

Respondents’ eligibility focused on their sexual behaviour and biological sex: people could participate if they had been assigned female biological sex at birth and had at least one female sexual partner^3^ (consensual same-sex sexual activity) in the past three years. This focus on sexual activity and biological sex avoided using pre-defined sexual and gender minority-related terms, implying an internalisation of specific personal identities. The aim was to look at sexual health risk behaviour and health-seeking behaviour. The focus on sexual behaviour rather than self-identified sexual orientation or gender identity aligns with the study's aim. The study's population consisted of people who identified as heterosexual, lesbian, bisexual, lesbian, gay, or queer, and queer womxn, gender-non-conforming people (assigned female biological sex at birth) and trans men.

For the qualitative data collection, participants were divided into three different groups. Group 1 was made up of queer womxn and trans men who had to meet the same eligibility criteria as the survey participants. Group 2 participants needed to be working with and for NGOs serving sexual and gender minorities. Group 3 was made up of key informants chosen based on their knowledge, background, and ability to speak on issues related to sexual and gender minority concerns, such as teachers, lawyers and policy experts.

### Data collection

**Fig. 1:**
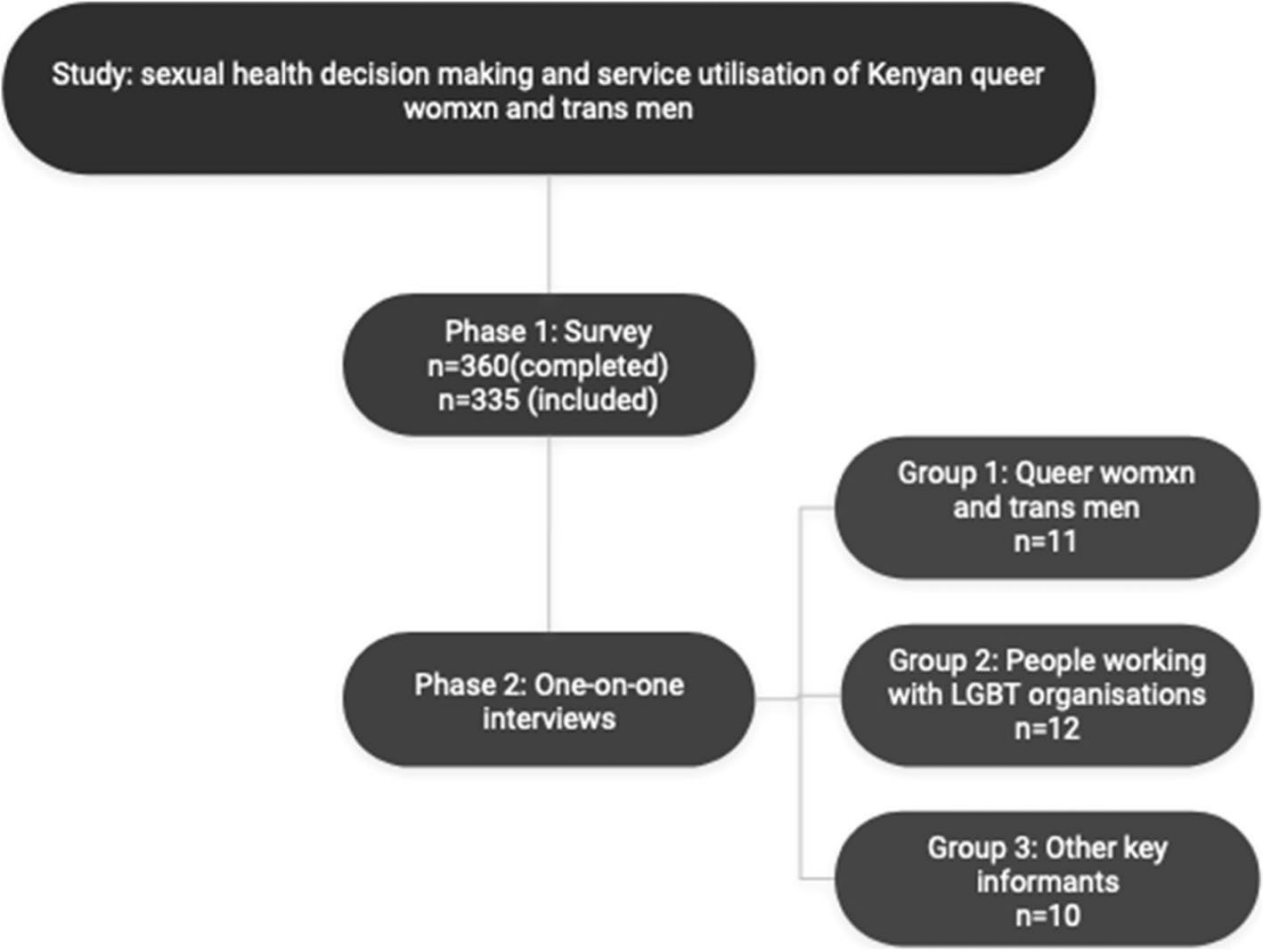
Data collection flow

Figure 1 shows the flow and stages of data collection. A survey was conducted first, followed by one-on-one interviews with participants from the three different groups.

#### Survey

Based on the literature findings and conceptual models, a survey was made available online viaREDCap, a secure tool for biomedical survey-based research. The aim was to reach 280 Kenyan queer womxn and trans men to identify their sexual health needs, knowledge, and risk. In order to reach the initial 280 respondents, three ‘influencers’ in the queer womxn and trans men communities were identified, who then shared the link to the survey within their networks. Influencers were defined as key figures in the community known to the researchers; they were considered people other queer womxn and trans men trusted and hence would trust messages shared by them.

Before dissemination, the survey was piloted with the influencers. The purpose of this was two-fold: first, to ensure that the survey was culturally and language-appropriate. Secondly, this enabled ‘buy-in’, as it was hoped that influencers might be more likely to share the survey if they approved and saw the relevance of its content and felt a sense of ownership through participation in the pilot. Once it was finalised, the link to the survey was distributed to all influencers who participated; three of whom then shared the survey within the queer womxn and trans men communities, mainly via WhatsApp groups.

Participants were compensated for their internet usage with a 200 Kenyan Shilling (approx. US$1.80) prepaid mobile data voucher. To discourage answering the survey multiple times, the informed consent form stated that participants should only participate once. A question on the survey asked how many times a participant had completed it; only participants who said they had not responded before could complete the survey. Participants were asked to create a unique identifier (their current or all-time favourite song and their mother's birth year). The choice for 'song' over other unique identifiers, such as digits of I.D. number or complete phone number, was to keep the participants’ identity anonymous. It should, however, be noted that multiple participations cannot fully be eliminated when conducting a self-administered online survey.

#### Interviews

In addition to the survey, one-on-interviews were conducted. Respondents were divided into three groups: the first group of participants, queer womxn and trans men themselves, were interviewed to get an in-depth understanding of the factors that shape the motivations, experiences, relationships and internal barriers that inform sexual health decision-making and service utilisation. The second group consisted of people working with and for organisations that serve Kenyan sexual and gender minorities, and queer womxn and trans men in particular. The questions explored the societal and community barriers that influence queer womxn and trans men’s behaviour. The last group were key informants, defined as people from various backgrounds and expertise that could assist in understanding how the established gaps could be addressed at a macro and micro level, explore whether structural solutions could be found, and assess their implementability.

The first group was sampled via the influencers and queer womxn and trans men who had participated in the survey and indicated wanting to be interviewed. The second and third groups were recruited through a convenience sample within the researchers’ professional network, combined with snowball sampling.

#### Safety

The participants’ safety was a key consideration when designing the quantitative and qualitative components of the data collection. Online spaces provide the opportunity to do this. The risks for the participants were assessed in detail for all aspects of the research, and mitigation measures were devised based on these risks. They included detailed information for the participants on staying safe online (Appendix 1) and a self-risk assessment (Appendix 2) for the survey, as well as conducting the interviews in safe, non-conspicuous spaces initially; over the COVID-19 pandemic, interviews were conducted on Skype and Zoom.

### Evaluation of the approach

**Fig. 2:**
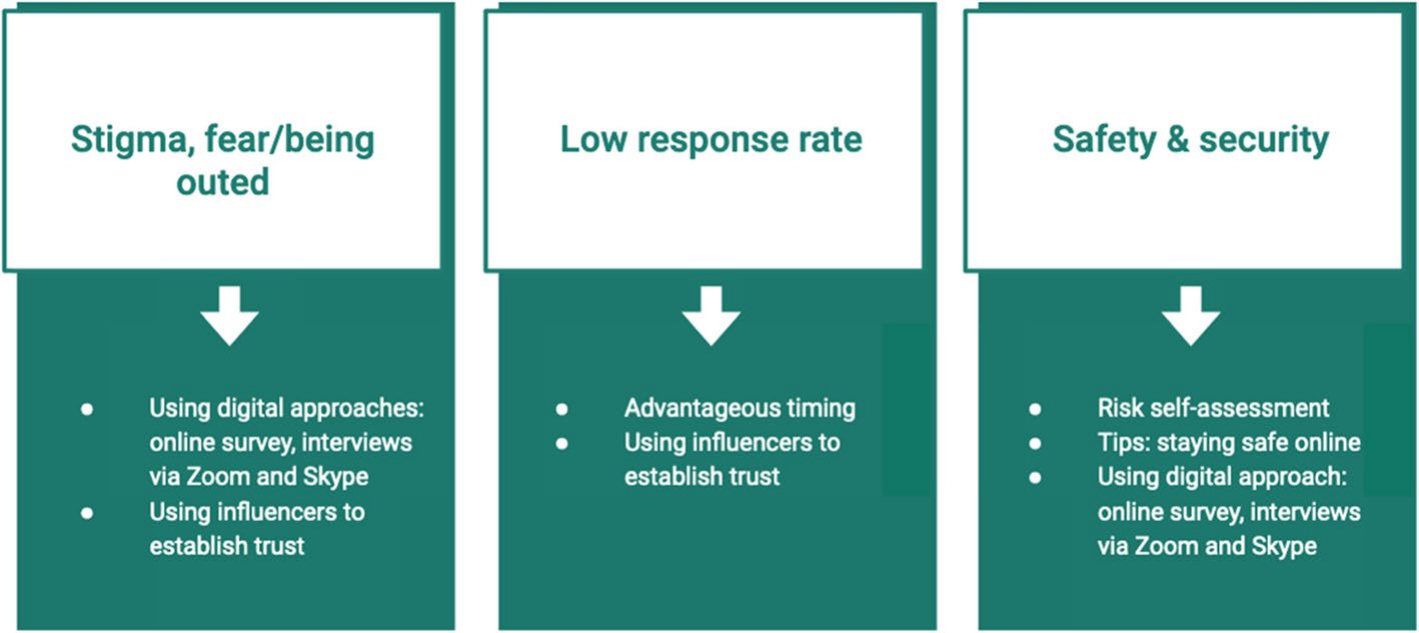
Risks and their mitigation

Four aspects were considered important to reach the desired sample successfully: using online spaces, having trusting relationships, timing, and safety and Security. Figure 2 shows the anticipated concerns from the literature review and how they were mitigated.

### Using online spaces to recruit and collect data

The research design and recruitment for the online survey were extremely successful.

 The survey was only shared with three influencers: members of the queer womxn and trans men communities known to the researcher, who shared the survey link within their networks, mainly via WhatsApp groups. All three had also participated in the pilot. ﻿﻿Within one day of sharing the survey, 260 people of the intended 280 had completed the survey. At this point, the survey was closed to evaluate the preliminary outcomes and get ethics committee approval to increase the sample size to *n* = 360.

Once approval for the increase of the sample size was obtained, approximately two weeks later, the influencers were asked to share the survey to target one sub-section of the underrepresented population in particular: trans men. After 14 days, the survey was closed again because 360 participants were reached. Of those, 335 were included in the research. Data from 17 participants were excluded because they had not provided full consent, and eight participants were excluded because they had extensive missing data.

For qualitative sampling, the approach was equally successful. Of the 36 people approached to participate in interviews, 33 agreed. Two people from the Group 3 (one healthcare provider of a family health NGO, the other a Youth SRHR Project Manager from the same NGO) stopped responding once they heard that the topic revolved around sexual and gender minorities. The third person (Group 2) never responded to the initial invitation email. Of the eleven Group 1 participants, eight had also been survey respondents and were contacted because they indicated their interest in being interviewed in this qualitative arm of the study.

It was important to have an in-depth understanding of online spaces and how they can best and safely be utilised. Kenya has always been a forerunner in mobile technology on the African continent and even worldwide, with high internet penetration and smartphone ownership [11]. Using online spaces for the survey hence seemed like a feasible approach. An additional advantage was that a wider variety of people from all over the country could be reached, an advantage identified in the literature [34].

Using video conferencing software made the qualitative data collection process more efficient and cost-effective. No travel time was needed from both the participants and the researcher's end, and no logistics measures to find safe spaces were necessary. A disadvantage of this approach was the exclusion of queer womxn and trans men without financial, physical or other access to the internet or the necessary hardware. Moreover, while video conferencing does allow for seeing facial expressions, a more holistic assessment of body language was lacking.

Bonevski et al. [6] raised concerns about higher research costs with hard-to-reach populations, especially if trusting relationships need to be established. As those relationships already existed, little effort was needed for this. Additionally, other costs for travel, space rental, refreshments, and others were not applicable by conducting the data collection online.

### Trusting relationship

Mistrust for the researcher can be a barrier in collecting data from sexual and gender minorities [18]. This is partly due to sexual orientations and gender identities other than heterosexuality and cisgender binaries having been considered psychopathological, and the negative impact on the perception and treatment of sexual and gender minorities in healthcare settings persists [7]. One of the main factors that positively influenced the success of the recruitment and data collection efforts was that the first author researcher had been working in the sexual and reproductive health and rights (SRHR) space in Kenya for over seven years at the point of piloting. Her work in SRHR information dissemination also had focused on the inclusion of vulnerable or marginalised populations, including sexual and gender minorities, populations that are often excluded in information dissemination efforts. These efforts were appreciated, and the researcher was able to build relevant relationships and develop trust with people within the queer womxn and trans men communities and establish relationships with organisations working for and with sexual and gender minorities. As the researcher identifies as a cisgender, heterosexual woman of European descent, having this support is assumed to have been very valuable and beneficial as an endorsement, no new relationships needed to be established, and an understanding of trust already existed. Working from this basis of trust, the researchers had fewer concerns from the participants’ side that they were being exploited for their stories and data. This confirms the research by Gatlin and Johnson [18].

The researchers were surprised by the positive responses and even gratitude expressed by the participants for being asked to be interviewed and sharing their opinions. Several survey respondents left positive remarks in the open comment section, such as, *‘I enjoyed taking the survey’* and *'Thank you so much for the wonderful survey'.* During the qualitative data collection, participants similarly voiced that they appreciated being part of the study and that they understood its relevance and necessity invalidating their needs for access to services and information, as well to remove restrictive legal sections from the Penal Code. *For many years, I can say since the existence of LGBTI movements in Kenya, the issue of LBQ women and trans men were never discussed. (…) it’s so awesome that you're now doing this. So needed. So awesome.’* (Trans man, Activist, Western Kenya).

It should be noted that trust is often expressed within binaries: participants either trust or distrust researchers. After months of fieldwork with vulnerable populations, a study argued that trust is a multi-layered phenomenon and that trust and distrust can coexist; researchers should be cognizant of this and examine the level of trust shown by participants [8]. In this research, it seems that the level of trust the participants had was high, that they trusted the direction of questioning and felt their opinions were validated. This may not have been due to the participants trusting the researcher and their intentions directly but rather due to the participants trusting the influencers and assessing the researchers’ trustworthiness, creating an additional level of trust. The participants may not have known the researchers, but the resulting multi-layered trust may positively affect the research outcomes.

While this research had not been intentionally designed to be community-participatory, the success indicates the importance of including community members in research, as also noted by other researchers [18, 37].

### Timing

The study's data collection took place when issues of sexual and gender diversity were highly publicised in mainstream and social media because of a court case to decriminalise same-sex activity. Two petitions [25]  were filed with the Kenyan High Court, Petition 150 and Petition 234, both of 2016, to repeal the sections of the Penal Code that criminalise consensual same-sex activities. The High Court rejected the petitions and found that the Penal Code should be upheld and criminalised same-sex activity. This High Court judgment was issued a few weeks before piloting started.

One of the reasons given by the court was that there was insufficient evidence for the discrimination of sexual and gender minorities in healthcare settings in Kenya:

*‘The Petitioners and the Interested Parties supporting the Petition argued that their right to health as stipulated in Article 43(1) had been violated. That may be true. However, no evidence was placed before this court to support the allegations. None of the Petitioners tendered evidence to prove that they had been denied medical attention in any health facility in the country or were subjected to mistreatment in the course of seeking medical attention. They merely made generalised statements without proof. Based on our analysis of the material placed before us, and this being a constitutional Petition, it is our conclusion that the answer to this issue is in the negative.'* [25 para. 308]

It emerged from the qualitative data collection that many sexual and gender minority communities disagreed with this assessment. They felt that the court had not considered the existing evidence and disregarded testimonies. During the data collection, it became evident that many Kenyan queer womxn and trans men wanted to be heard and share their stories: this was mentioned by many people, both in the quantitative and qualitative data collection. For example, a respondent to the survey said, ‘*Kindly use this information in the Repeal 162 [the court case in question] so that the court can have concrete evidence about our discrimination*’.

The High Court’s decision was mentioned in most interviews, especially among queer womxn and trans men and people working for LGBT-focused organisations. There was a great disappointment but also hope that the appeal to the court’s decision would be successful and that more data would play a role in informing future rulings. With the High Court’s decision on participants’ minds, there was high interest in the research topic. Thus, a lack of interest, identified as a barrier for researching hard-to-reach populations by other researchers [18], was not a concern for this study. It should be noted that this research timing was not planned with the date of the High Court ruling in mind, as the court case had been postponed multiple times. The timing was hence a serendipitous event.

### Safety and security

In order to mitigate any safety risks for the participants of both the quantitative and qualitative data collections, several measures were put in place. Firstly, the survey participants were provided with an information sheet on how to stay safe online (information on creating safe passwords and protecting them, browser history setting, using virtual private networks). Secondly, contact details for sexual and gender minority-friendly organisations were provided if the participants had any questions or concerns about their sexual or mental health or required legal support. Finally, participants were asked to complete a self-assessment about potential individual risks, which intended to prompt their reflection on risk and support them in making the most informed decision possible about whether or not to participate. If they identified themselves to be at high risk, they were explicitly asked if they wanted to participate despite that risk. Not only did did this, in combination with the online approach, ensure the anonymity of the participants, as they were able to complete the survey on their phones or computers from the privacy of their own spaces [34], it also reduced the risk of being ‘outed’, as this meant not having to go to spaces associated with sexual and gender minorities or to be seen with a sexual health researcher. Russomanno, Patterson, and Tree (2019) also stress the importance of having detailed risk analyses and safety measures when conducting research using online tools [42].

The biggest initial concern was the safety of the participants during the qualitative data collection. The interviews were to be conducted in safe spaces. The primary space was going to be at a centrally located music academy, as their studios were sound-proof, and there was hence no risk of being overheard. Also, the spaces were not associated with sexual and gender minorities. However, only the first eight interviews were held in person; five were queer womxn identified through the survey and three were Group 3 (key informants). Due to the COVID-19 pandemic that started to significantly affect the lives of Kenyans starting mid-March 2020, the researcher stopped all in-person interviews and instead conducted them over Zoom and Skype, and in two cases, over the phone due to connectivity issues. Not only did this take some of the logistics issues of finding safe spaces away, but participants also did not need to travel or worry about the accessibility or safety of the chosen spaces: participants could do interviews from the safety of their own homes. There was no risk of being overheard and no risk of being seen with an LGBT researcher or being seen or associated with LGBT organisations. As videos were used during the interviews, concerns such as a lack of body language that could be a problem during phone interviews were not a problem.

It should be stressed that whether or not this recruitment and sampling approach using influencers in online settings can hence be successful will depend on the setting and context and other factors, such as the ability of the researcher to establish trust with the participants.

### Limitations

As the snowballing started with only three influencers, people who were not associated with some part of the queer womxn and trans men networks were unlikely to be sampled, hence introducing bias, a concern about online recruitment also mentioned by McInroy [34]. Because the study's aim was not to recruit a representative sample, this was considered acceptable, but it posed an important limitation to the generalisability of the survey’s findings. As was found in a 2014 U.S. study [20], the online approach may disproportionately reach younger and highly educated people: of survey respondents, 85% were under the age of 35, were highly educated,56% had either attended some university or completed their university education. Due to high levels of education and tech-understanding of the sample, they possibly represented queer womxn and trans men with better knowledge and agency than other Kenyan sexual and gender minority people. Digital literacy could be a barrier to research participation, which could hold true for other populations, as Singh et al. (2021) note. While there was variance in geography, age, and other demographics of the sample population, other sub-communities, such as very rural people, less educated people, those without access to the necessary technology were excluded as they were not within the influencers' networks or may not have had trust in the research [52]. Additionally, it is also possible that health status and service needs will change over their lifespans and that this was not adequately captured with this research.

An important determinant of people's access to using the internet on their mobile phones is access to legal SIM cards. In Kenya, valid identity documentation is necessary to purchase a SIM card. This requirement can be a barrier to reaching populations who do not possess legal identification. The United Nations High Commissioner for Refugees (UNHCR) has pointed this out for research with refugees considered a hard-to-reach population [45]. It also is a crucial consideration for reaching trans and gender diverse people. Access to legal gender recognition is possible but difficult in Kenya. Many trans and gender diverse people do not have identity documents that correctly reflect their gender identity and expression as the processes of changing legal documents with gender markers are unclear [10]. Buying a SIM card with such a non-matching identity document (ID) means that trans people risk being outed (because the sex recorded on their ID does not correspond to their gender identity) or be accused of fraud if it is assumed that the ID document belongs to someone else [38, 10]. People without legal documentation may have thus been excluded from this research.

Care should be taken not to marginalise those who do not have access to technology or the internet further, which means that, in some instances, online approaches will not be appropriate. This is thus the study's main limitation; Kenyan queer womxn and trans men who do not have access to technology and the internet or were outside out of networks that the influencers could reach could not contribute to the study.

## Conclusion

For the purpose of this research on Kenyan queer womxn and trans men and how they make decisions regarding sexual health and service utilisation, recruitment, and data collection using online spaces was successful.

Respondents for the survey were identified and recruited more rapidly than anticipated, which was due to several factors: the existing, trusting relationships the researcher had with key people in the population to be studied; buy-in from influential and well-connected community members who identified as LGBTQI + people themselves; interest and enthusiasm for the topic at hand; the timing of the survey immediately following a high-profile court case, and thorough online safety precautions. Concerns around stigma and violence were mitigated by participants completing the survey or participating in interviews online, hence lowering barriers to participation, such as fear of being seen with the researcher or other queer womxn and trans men.

Some of the positive outcomes of the survey had not been anticipated in the original study design. Additionally, concerns around safety and security during qualitative data collection were mitigated by using video conferencing tools. Although this was initially done due to the COVID-19 pandemic, it proved to be an effective and safe way to collect sensitive data. It also reduced the need to find safe places and the time needed for interviews, as commuters were not needed.

Disadvantages, such as low response rates, the inability to walk participants through the survey, posing an annoyance and low external validity, as described by Kılınç and Fırat [27], was not found. A low response rate was not a concern in this case. It is unknown whether people who received the link multiple times from various sources may have felt bothered by it, if so, it was not brought to the researchers’ attention. Generalisability is certainly a concern for the outcomes of this research, which is amplified by how little is known about the population in general. Future research needs to be conducted in order to be able to make more generalised conclusions. Not being able to explain survey questions in more depth to participants was also a disadvantage in this study.

The confluence of factors – trusting relationships, timing, focus on safety and security – worked well for this research. It might work well in other settings, where data collection with marginalised communities might not be possible, and sampling might be difficult, or participants could be at risk. Including key community members in data collection efforts proved important and effective, underscoring the need to do community-participatory research when designing research projects centred around marginalised communities. While the timing of the research and the High Court ruling were serendipitous, other events or developments that can galvanise interest can be purposively addressed in research design.

Including sexual and gender minorities and other marginalised populations in research is important to ensure an in-depth understanding of their health needs, vulnerabilities, and barriers to seeking care. Such knowledge should be considered in health policy making and service planning, to ensure that ‘no-one is left behind’. In turn these should positively impact overall health and well-being in underserved communities.

### More in-depth research is needed

While the findings of this research are an important start to getting a better understanding and more knowledge of the needs of sexual and gender minorities in Kenya, and specifically queer womxn and trans men, more exploration is needed, with special attention to a broader representation in the sampling, as well as a reduction of the bias that could have been introduced by using the described snowballing techniques. Continuous efforts need to be made to include sexual and gender minorities in research efforts. While efforts for improvement are ongoing, recent developments to exclude and even erase sexual and gender minorities from data scans in federal programs in countries, as is the case in the United States, need to be vigilantly monitored, as ‘*data sets that do not include sexual orientation and gender identity information are inadequate and incomplete*’ ([30], p. 2).

Continuous efforts need to be made globally to advocate for the inclusion of sexual and gender minorities and other hard to reach populations in research to better understand needs, barriers and other factors that could influence health and well-being, as well as the careful design of interventions to address inequities so that ‘no-one should be left behind’.

## Data Availability

Due to the sensitive nature of the dataset, data will not be made available. The information on staying safe online shared with the participants can be made available from the corresponding author on reasonable request.

## Appendix 1

### Risk self-assessment

In this survey, you will be asked personal questions about your sex life, your mental and emotional health, about alcohol or drug use, and about potentially criminal behaviour, because some same-sex activities are illegal in Kenya.

All information you give is confidential. Note: If you are visually impaired, the use of reading apps or assistive devices can cause a lack of privacy. Some of these topics could make you feel uneasy or bring up negative emotions. Some questions you can skip if they are unsettling to you. Other questions need to be answered. If they make you uncomfortable, you can stop taking part at any time. Before you get started with the survey, please answer the questions below and carefully read through the tips to keep you safe online.

**Table:**

	Yes	No	I don’t know
Do you think answering questions about sexuality, sexual health, drug and alcohol use and mental health could cause you any kind of stress?			
Have you been advised by a healthcare professional to refrain from discussing emotional issues you are dealing/have dealt with?			
Do you think you could be physically harmed if anybody found out that you participated in this research?			
Do you think you could be emotionally harmed if anybody found out that you participated in this research?			
Do you think you can face any legal consequences if anybody found out that you participated in this research?			

If participants **i**ndicated ‘yes’ or ‘I don’t know’ in any of the questions above:

**Table:**

	Yes	No
You said this research may put you at risk. Do you still want to participate?		

Finally, if any issues come up during the survey, you can either contact the researcher via email or one of the following organisations for assistance.

If you have questions about HIV testing, treatment, and other concerns, please get in touch with LVCT Health or their one2one hotline (800 720 121 or 1190, both toll-free) for sexual health questions. For questions around abortion, safe sex, and birth control, please get in touch with Marie Stopes Kenya. For mental health and problems with drugs and alcohol, please head to this sexual and gender minority specialised online counselling service. In case of any legal issues you may face in your private life, the National Gay and Lesbian Human Rights Commission provide legal help to sexual and gender minority people.

## Appendix 2

### Staying safe online information

We want you to stay safe while participating in this study. If you keep your devices safe, there will be minimal risk that someone finds out you took part and could ‘out’ you against your will. Here are some tips.

#### Passwords and pins

Passwords and pin numbers are really important your safety, especially when you are sharing devices. Make sure all your devices have random passwords with letters, numbers, and special characters. Sentences work well, too. The more difficult, the better. Use a different password for every device and each platform/e-mail address/website you use. That will make it easier for you to keep your privacy, even when your computer is hacked or your phone is stolen.

Make sure you log out of accounts when you are done with your session.

Software like 'KeePass’ make it easier for you to have and store complicated passwords.

#### Clearing your browser history

Clearing your browser history is especially important when other people use the same devices as you, but you should regularly do it if you visit websites etc. that could tell people about your sexual orientation or gender identity. To check how to clear your browsers history, click here.

#### Using VPNs

If you are worried about anyone tracking you online, you can use a 'VPN' (Virtual Private Network). A VPN helps you get your information safely to and from your computer (encryption), and it hides your real location, so you can't be traced, allowing you to surf anonymously.

To use a VPN, download the Psiphon app to your iPhone or Android smartphone, click ‘connect’ and nobody will be able to tell where you are browsing from. On a desktop, apps like betternet work the same way.

#### Shared devices

Staying safe when sharing devices with friends, colleagues, family, or at an internet café can be a problem. Make sure nobody can watch your screen. Clear your browsing history when you are done, and don’t forget to log out of any social media sites or accounts you may have used.

#### Malware and hacker protection

Hackers can attack your computer and steal your information. Make sure that you have an antivirus programme installed, a firewall activated, and never open email attachments from people you don't know and trust. If your phone or computer are acting strangely, the battery dies more quickly than usual, and they get very hot, someone may be monitoring your device.

If you want to know more about staying safe online, have a look at Tactical Tech’s ‘Security in a Box’ information.

## Abbreviations

LGBT: Lesbian, gay, bisexual, transgender
LGBTQ: Lesbian, gay, bisexual, transgender, queer
MSM: Men who have sex with men
NGO: Non-governmental organisation
SRHR: Sexual and reproductive health and rights
STI: Sexually transmitted infections

## Footnotes

1 The participants indicated preferring the terms queer and womxn when referring to themselves; womxn is an explicitly inclusive intersectional feminist term.

2 ‘*People who do not wish to be found or contacted’* [18].

3 Self-identified or identified/perceived as female by the study participants.

## References

[1] Allen L (2015). Queering the academy: new directions in LGBT research in higher education. High Educ Res Dev.

[2] Amnesty International. Crimes of hate, conspiracy of silence. Torture and ill-treatment based on sexual identity. [Internet]. Amnesty International; 2001. Available from: https://www.amnesty.org/download/Documents/120000/act400162001ar.pdf

[3] Arbeit M, Fisher C, Macapagal K, Mustanski B (2016). Bisexual Invisibility and the Sexual Health Needs of Adolescent Girls. LGBT Health.

[4] Babyar J (2018). Equitable health: let’s stick together as we address global discrimination, prejudice and stigma. Arch Public Health..

[5] Blondeel K, de Vasconcelos S, García-Moreno C, Stephenson R, Temmerman M, Toskin I (2017). Violence motivated by perception of sexual orientation and gender identity: a systematic review. Bull World Health Organ.

[6] Bonevski B, Randell M, Paul C, Chapman K, Twyman L, Bryant J (2014). Reaching the hard-to-reach: a systematic review of strategies for improving health and medical research with socially disadvantaged groups. BMC Medical Research Methodology..

[7] Byne W (2014). Forty Years After the Removal of Homosexuality from the DSM: Well on the Way but Not There Yet. LGBT Health.

[8] Celestina M (2018). Between trust and distrust in research with participants in conflict context. Int J Soc Res Methodol.

[9] Cochran S, Mays V (2017). Advancing the LGBT Health Research Agenda: Differential Health Trends Within the Lesbian, Gay, and Bisexual Populations. Am J Public Health.

[10] Chiam Z, Duffy S, González Gil M, Timothy N, Patel M. Trans legal mapping report. Recognition before the law [internetinternet]. ILGA; 2021. Available from: https://ilga.org/downloads/ILGA_World_Trans_Legal_Mapping_Report_2019_EN.pdf

[11] Communications Authority of Kenya. Second-quarter sector statistics report for the financial year 2017/2018. [Internet]. Communications Authority of Kenya; 2018. Available from: https://ca.go.ke/wp-content/uploads/2019/03/Sector-Statistics-Report-Q2-2018-19.pdf

[12] D’Augelli A, Grossman A, Starks M. Families of Gay, Lesbian, and Bisexual Youth. J GLBT Fam Stud. 2008;4(1):95–115.

[13] D’Augelli A, Hershberger S, Pilkington N. Lesbian, gay, and bisexual youth and their families: Disclosure of sexual orientation and its consequences. Am J Orthopsychiatry. 1998;68(3):361–71. 10.1037/h0080345 9686289

[14] Eliason M, DeJoseph J, Dibble S, Chinn P (2012). LGBT Health Research: Introduction to the Special Issue. Journal of Homosexuality.

[15] Fisher C, Mustanski B (2014). Reducing Health Disparities and Enhancing the Responsible Conduct of Research Involving LGBT Youth. Hastings Cent Rep.

[16] Fisher C, Wallace S (2000). Through the Community Looking Glass: Reevaluating the Ethical and Policy Implications of Research on Adolescent Risk and Psychopathology. Ethics Behav.

[17] Gay and Lesbian Coalition of Kenya. Research on the lived experience of lesbian, bisexual, and queer women in Kenya. [Internet]. 2016. Available from: https://www.galck.org/wp-content/uploads/2021/03/Research-on-the-lived-experiences-of-LBQ-women-in-Kenya.pdf

[18] Gatlin T, Johnson M. Two Case Examples of Reaching the Hard-to-Reach: Low Income Minority and LGBT Individuals. Journal of Health Disparities Research and Practice [Internet]. 2017 [cited 9 August 2021];10(3). Available from: https://digitalscholarship.unlv.edu/jhdrp/vol10/iss3/11

[19] Harper G, Bruce D, Serrano P, Jamil O. The Role of the Internet in the Sexual Identity Development of Gay and Bisexual Male Adolescents. The Story of Sexual Identity. 2009;:297–326

[20] Heen M, J.D L, T.D M (2014). A Comparison of Different Online Sampling Approaches for Generating National Samples. UNLV Center for Crime and Justice Policy.

[21] Hoebel J, von der Lippe E, Lange C, Ziese T (2014). Mode differences in a mixed-mode health interview survey among adults. Archives of Public Health..

[22] Human Rights Watch. This Alien Legacy. The origins of ‘sodomy’ laws in British Colonialism. [Internet]. 2008. Available from: https://www.hrw.org/report/2008/12/17/alien-legacy/origins-sodomy-laws-british-colonialism

[23] Integrated Sexual Orientation Gender Identity and Expression Community Online Platform (ICOP). Kenya HIV Prevention Prioritization Evidence Note. [Internet]. 2014. Available from: http://www.icop.or.ke/wp-content/uploads/2016/09/Kenya-HIV-Prevention-Prioritization-evidence-note-11th-June-2014.pdf

[24] Kenyan Human Rights Commission. The outlawed amongst us. [Internet]. 2011. Available from: https://www.khrc.or.ke/mobile-publications/equality-and-anti-discrimination/70-the-outlawed-amongst-us/file.html

[25] Petition 162 &176 (Consolidated). 2019.

[26] Kenya National Commission of Human Rights (KNCHR). Realising Sexual and Reproductive Health Rights in Kenya: a myth or reality? [Internet]. 2012. Available from: https://www.law.utoronto.ca/utfl_file/count/documents/reprohealth/kenya_reproductive_health_report.pdf

[27] Kılınç H, Fırat M (2017). Opinions of Expert Academicians on Online Data Collection and Voluntary Participation in Social Sciences Research. Educational Sciences: Theory & Practice..

[28] Lange A, Duran A, Jackson R (2019). The State of LGBT and Queer Research in Higher Education Revisited: Current Academic Houses and Future Possibilities. J Coll Stud Dev.

[29] Laws of Kenya. The Penal Code. National Council for Law Reporting; 2009.

[30] Loewy K (2017). Erasing LGBT People From Federal Data Collection: A Need for Vigilance. Am J Public Health.

[31] Martin J, Meezan W. Applying Ethical Standards to Research and Evaluations Involving Lesbian, Gay, Bisexual, and Transgender Populations. Handbook of Research with Lesbian, Gay, Bisexual, and Transgender Populations. 1st ed. Abingdon: Taylor & Francis Group; 2008.

[32] Mathiesen K (2012). The Human Right to Internet Access: A Philosophical Defense. The International Review of Information Ethics.

[33] McCormack M (2014). Innovative sampling and participant recruitment in sexuality research. J Soc Pers Relat.

[34] McInroy L (2016). Pitfalls, Potentials, and Ethics of Online Survey Research: LGBTQ and Other Marginalised and Hard-to-Access Youths. Social Work Research.

[35] Meyer I, Wilson P (2009). Sampling lesbian, gay, and bisexual populations. J Couns Psychol.

[36] Minority Women in Action. Breaking the silence. The status of women who have sex with women in Kenya. [Internet]. Nairobi; 2013. Available from: https://www.galck.org/wp-content/uploads/2017/01/Breaking-the-Silence-Status-of-Kenyan-WSW-2013-first-version.pdf

[37] Molyneux S, Sariola S, Allman D, Dijkstra M, Gichuru E, Graham S (2016). Public/community engagement in health research with men who have sex with men in sub-Saharan Africa: challenges and opportunities. Health Research Policy and Systems..

[38] Müller A. Briefing Document: Legal Gender Recognition in Botswana. [Internet]. 2020. Available from: http://www.southernafricalitigationcentre.org

[39] Mustanski B (2011). Ethical and Regulatory Issues with Conducting Sexuality Research with LGBT Adolescents: A Call to Action for a Scientifically Informed Approach. Arch Sex Behav.

[40] Mustanski B, Lyons T, Garcia S (2010). Internet Use and Sexual Health of Young Men Who Have Sex with Men: A Mixed-Methods Study. Arch Sex Behav.

[41] Mustanski B (2001). Getting wired: Exploiting the internetinternet for the collection of valid sexuality data. J Sex Res.

[42] Russomanno J, Patterson J, Jabson Tree J (2019). Social Media Recruitment of Marginalized, Hard-to-Reach Populations: Development of Recruitment and Monitoring Guidelines. JMIR Public Health and Surveillance..

[43] Savin-Williams R (2009). The New Gay Teenager.

[44] UHAI-EASHRI. A people condemned. The Human Rights Status of lesbian, gay, bisexual, transgender and intersex persons in East Africa: 2009–2010. Nairobi: UHAI-EASHRI; 2010.

[45] United Nations High Commissioner for Refugees (UNHCR). Displaced & Disconnected: Connectivity for Refugees [Internet]. Geneva; 2019. Available from: https://www.unhcr.org/innovation/wp-content/uploads/2019/04/Displaced-Disconnected-WEB.pdf

[46] van Eeden-Moorefield B, Few-Demo A, Benson K, Bible J, Lummer S (2017). A Content Analysis of LGBT Research in Top Family Journals 2000–2015. J Fam Issues.

[47] Waite S, Denier N. A Research Note on Canada’s LGBT Data Landscape: Where We Are and What the Future Holds. Canadian Review of Sociology/Revue canadienne de sociologie. 2019;56(1):93–117. 10.1111/cars.12232 30793865

[48] Weiss J (2011). Reflective Paper: G.L. Versus B.T.: The Archaeology of Biphobia and Transphobia Within the U.S. Gay and Lesbian Community. Journal of Bisexuality.

[49] Winter S, Diamond M, Green J, Karasic D, Reed T, Whittle S (2016). Transgender people: health at the margins of society. The Lancet.

[50] Karel, Blondeel Lale, Say Doris, Chou Igor, Toskin Rajat, Khosla Elisa, Scolaro Marleen, Temmerman (2016) Evidence and knowledge gaps on the disease burden in sexual and gender minorities: a review of systematic reviews. Int J Equity in Health 15(1):10.1186/s12939-016-0304-1. 10.1186/s12939-016-0304-1 PMC4724086 26800682

[51] Blondeel Lale K., Say D., Chou D., Toskin I., Khosla R., Scolaro E., Temmerman, M. (2016) Evidence and knowledge gaps on the disease burden in sexual and gender minorities: a review of systematic reviews. Int J Equity in Health 15(1):10.1186/s12939-016-0304-1. 10.1186/s12939-016-0304-1 PMC4724086 26800682

[52] Singh N.S., Lokot M., Chi-Chi U., Onyango M.A., Morgan R., Harmer A., Freedman J., Heidari S. (2021) Research in forced displacement: guidance for a feminist and decolonial approach. Lancet 397(10274):560–2. 10.1016/S0140-6736(21)00024-6. 10.1016/S0140-6736(21)00024-6 PMC9753228 33581808

